# Determination of Aflatoxin M_1_ in Milk by ELISA Technique in Mashad (Northeast of Iran)

**DOI:** 10.5402/2012/121926

**Published:** 2012-08-23

**Authors:** Ali Mohamadi Sani, Mohamad Khezri, Halime Moradnia

**Affiliations:** ^1^Department of Food Science & Technology, Quchan Branch, Islamic Azad University, Quchan Branch, Quchan, Iran; ^2^Quality Control Laboratory of Food & Drug Administration, Mashhad University of Medical Sciences, Khorasan, Mashhad 77558 3918, Iran

## Abstract

The aim of this study was to detect the amount of aflatoxin M_1_ (AFM_1_) in pasteurized milk samples in Mashad in northeast of Iran. For this purpose, 42 milk samples were collected from retail stores during fall 2011 and analyzed for AFM_1_ by enzyme-linked immunosorbent assay (ELISA) technique. All the analyses were done twice. Results showed presence of AFM_1_ in 97.6% of the examined milk samples by average concentration of 23 ± 16 ppt and contamination level ranging between 6 and 71 ppt. The concentration of AFM_1_ in all the samples was lower than the Iranian national standard and Food and Drug Administration limits (500 ppt), and, only in 3 (1.6%) samples, AFM_1_ concentration was more than the maximum tolerance limit (50 ppt) accepted by European Union and Codex Alimentarius Commission. According to our findings and previous studies, AFM_1_ contamination of milk is not a concern in this region, and the regional standard of AFM_1_ contamination in milk might be changed to lower than 100 ppt.

## 1. Introduction

Mycotoxins are secondary metabolites of molds which are associated with certain disorders in animals and humans. In addition to being acutely toxic, some mycotoxins are now linked with the incidence of certain types of cancer, and it is this aspect which has evoked global concern over feed and food safety, especially for milk and milk products [1]. Aflatoxin M_1_ (AFM_1_) is a hepatocarcinogen found in milk of animals that have consumed feeds contaminated with aflatoxin B1 (AFB1), the main metabolite produced by fungi of the genus *Aspergillus,* particularly *A. flavus, A. parasiticus,* and *A. nomius* [2]. About 0.3–6.2% of AFB1 in animal feed is transformed to AFM_1_ in milk [3]. Due to serious health concerns, many countries have set maximum limits for aflatoxins, which vary from country to country [4]. The European Community prescribes that the maximum level of AFM_1_ in liquid milk should not exceed 50 ppt. However, according to the US standard, the level of AFM_1_ in liquid milk should not be higher than 500 ppt [5]. There have been several studies on AFM_1_ concentration in milk samples in different regions of the world and also in Iran, but this study was done to evaluate the occurrence of AFM_1_ in milk distributed in Mashad in northeast of Iran in order to evaluate the potential of changing the regional standard on AFM_1_ contamination of milk.

## 2. Materials and Methods

### 2.1. Materials

#### 2.1.1. Samples

In this study the AFM_1_ content of pasteurized milk samples in retail stores in Mashad (northeast of Iran) was determined in fall 2011. Forty-two pasteurized milk samples (1000 mL milk packets, heat treated at 72–74.4Ć for 15–20′′) from different brands were collected by simple random sampling method. The samples were transported to the laboratory in an insulated container at about 4°C and analyzed upon arrival.

#### 2.1.2. Reagents

Most of the reagents used to detect AFM_1_ were contained in the RIDASCREEN test kit, which included microtiter plate coated with capture antibodies, AFM_1_ standard solutions used for the construction of the calibration curve (1.3 mL each 0, 5, 10, 20, 40, and 80 ppt), peroxidase-conjugated AFM_1_, substrate (urea peroxidase), chromogen (tetramethylbenzidine), and stop reagent contains 1N sulphuric acid. Methanol used was of analytical grade and provided by Merck.

### 2.2. Methods

#### 2.2.1. AFM_1_ Detection

The quantitative analysis of AFM_1_ in pasteurized milk samples was performed by competitive ELISA (RIDASCREEN AFM_1_, R-Biopharm) procedure as described by R-biopharm GmbH [6]. Prior to analysis of the samples, the ELISA method was validated to ensure data quality. Validation of ELISA was carried out by determination of recoveries and the mean variation coefficient for fresh milk spiked with different concentrations of AFM_1_ (5, 10, 20, 40 and 80 ppt). The results are expressed in Table 1.

Milk samples were centrifuged at 3500 g for 10 min at 10°C. The upper creamy layer was completely removed by aspirating through a Pasteur pipette and from the lower phase (defatted phase) 100 *μ*L was directly used per well in the test. One hundred *μ*L of the AFM_1_ standard solutions (100 *μ*L/well) and test samples (100 *μ*L/well) in duplicate were added to the wells of microtiter plate and incubated for 60 min at room temperature in the dark. After the washing steps, 100 *μ*L of the enzyme conjugate was added and incubated for 60 min at room temperature in the dark. The washing step was repeated three times. Fifty *μ*L of substrate and 50 *μ*L of chromogen were added to each well and mixed thoroughly and incubated for 30 min in the dark. Following the addition of 100 *μ*L of the stop reagent to each well, the absorbance was measured at 450nm in ELISA reader (ELX-800, Bio-Tek Instruments, USA). According to the RIDASCREEN kit guidelines, the lower detection limit is 5 ppt for milk.

#### 2.2.2. Evaluation of AFM_1_

**Figure 1:**
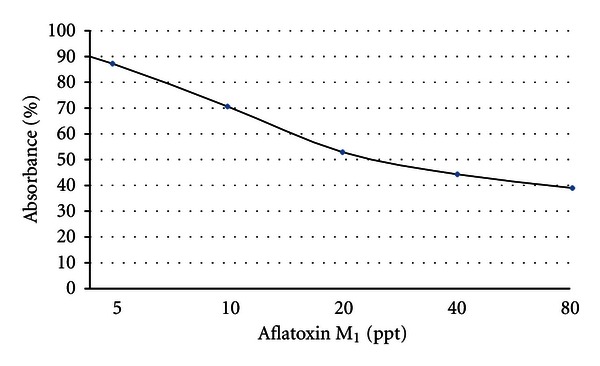
Calibration curve of standard solutions of AFM_1_ with concentrations of 5, 10, 20, 40, and 80 ppt by ELISA analysis.

The absorbance values obtained for the standards and the samples were divided by the absorbance value of the first standard (zero standards) and multiplied by 100 (percentage maximum absorbance). Therefore, the zero standard is thus made equal to 100%, and the absorbance values are quoted in percentages. The values calculated for the standards were entered in a system of coordinates on semilogarithmic graph paper against the AFM_1_ concentration in ppt (Figure 1). The equation of the trendline in Figure 1 is as follows:

(1) $$\begin{array}{c} y=0.016x^{2}-1.940x+91.34. \end{array}$$

## 3. Statistical Analysis

Data were analysed using Excel 2007 and results reported as mean ± SD. The calibration curve and trendline equation prepared using Excel 2007.

## 4. Results and Discussion

The standard solutions of concentration from 5 to 80 ppt AFM_1_ were used to find calibration/standard curve. The results showed the linearity of the standard curve over the range studied. Figure 1 gives the calibration curve of standard solutions of AFM_1_ with concentrations of 5, 10, 20, 40, and 80 ppt by ELISA analysis.

Analytical results showed that the incidence of AFM_1_ contamination in pasteurized milk samples was low. Although 97.6% of the samples were contaminated with AFM_1,_ the toxin concentration was lower than Iranian national standard and FDA limit (500 ppt) and only in three (1.6%) of the samples AFM_1_ concentration was greater than the maximum tolerance limit (50 ppt) accepted by European Union and Codex Alimentarius Commission.  Table 2 shows the distribution and percentage of AFM_1_ contamination in pasteurized milk samples. The minimum and maximum contamination level of AFM_1_ was found to be 6.4 and 71.4 ppt, respectively. The mean ± SD AFM_1_ level in the analyzed samples of pasteurized milk was 23 ± 16 ppt.

The mean AFM_1_ concentrations in milk in European, Latin American, and Far Eastern diets have been reported by the Joint FAO/WHO Expert Committee on Food Additives [7] to be 23, 22, and 360 ng/L, respectively. Thus, the observed mean AFM_1_ concentration in Mashad milk samples was as high as the European and Latin American and much lower than those reported for the Far Eastern diets.

On the other hand, several studies have been done to determine AFM_1_ contamination of milk in Iran (Table 3). The incidence of AFM_1_ observed in the present study was lower than the incidence of AFM_1_ reported by other authors [8–17], yet, in all studies, the averages of toxin concentrations are below 100 ppt. The variations may be attributed to differences in region, season, and especially analysis method.

Based on the above results, especially later studies in Mashad, the present situation is hopeful and might represent the possibility of altering standard limit of AFM_1_ concentration in milk in Iran. We suggest reduction of the limit as low as 100 ppt for raw milk.

## Figures and Tables

**Table 1:** Validation data of the competitive ELISA for AFM_1_.

AFM_1_ spiked (ppt) (*n* = 5)	AFM_1_ found (ppt)	Recovery (%)	Variation coefficient (%)
5	5	100	0.0
10	9.98	99.8	0.2
20	20.11	100.55	0.5
40	39.84	99.60	0.4
80	79.90	99.87	0.1

**Table 2:** Aflatoxin M1 distribution and percentage of pasteurized milk samples.

AFM_1_ levels ppt in positive samples					
	<10	10–30	30–50	50–70	>70*
*N* ^1^	5	27	5	3	1
%^2^	12.3	65.6	12.3	7.4	2.4

*N* ^1^ number of contaminated samples.

%^2^ Percentage of AFM_1_ positive samples.

^∗^71.3 ppt in the contaminated sample.

**Table 3:** The incidence of milk contamination in Iran in other studies.

Location	Reference	Method of detection	Sample size	Percent of contamination	Percent of contamination >50 ppt	AFM_1_ concentration (ppt)
Mashad (north east of Iran)	Current study	ELISA	42	97.6	1.6	23.2
Mashad (north east of Iran)	Mohamad Sani and Nikpooyan, 2012 [8]	HPLC	60	100	1.6	16.16
Mashad (north east of Iran)	Mohamadi Sani et al., 2010 [9]	ELISA	196	100	80.6	77.9
Five states of Iran	Tajkarimi et al., 2007 [10]	HPLC	98	100	37.7	39
Tehran (capital of Iran)	Heshmati and Milani, 2010 [17]	ELISA	210	55.2	33.3	58
14 states of Iran	Tajkarimi et al., 2008 [13]	HPLC	319	54	23	57
Shiraz (south of Iran)	Alborzi et al., 2006 [12]	ELISA	624	100	17.8	n.r*
Ahwaz (south of Iran)	Rahimi et al., 2010 [16]	ELISA	311	42.1	12.5	43.3
Sarab (north west of Iran)	Kamkar 2005 [11]	TLC	111	76.6	40	61.4
Central part of Iran	Fallah 2010 [14]	ELISA	225	67.1	33.1	49.9
Ardabil (north west of Iran)	Nemati et al., 2010 [15]	ELISA	90	100	33	n.r*

^∗^Not reported.
